# The Alkane 1‐Monooxygenase Gene *alkB* of *Pseudomonas* sp. FF2 Is Upregulated During Colonisation of *Arabidopsis thaliana* Leaves

**DOI:** 10.1111/1758-2229.70242

**Published:** 2025-11-24

**Authors:** Rudolf Schlechter, Laura Voß, Evan J. Kear, Mila Oeltjen, Mitja Remus‐Emsermann

**Affiliations:** ^1^ Department of Biology, Chemistry, Pharmacy, Freie Universität Berlin Institute of Microbiology and Dahlem Centre of Plant Sciences Berlin Germany

**Keywords:** alkane metabolism, bacterial adaptations, bioreporter, green fluorescent protein, single‐cell heterogeneity

## Abstract

Bacteria on leaf surfaces encounter variable access to nutrients and water. This oligotrophic environment is partly due to cuticular waxes that render the leaf surface hydrophobic. While the alkane hydroxylase gene *alkB* is widespread in leaf‐associated bacteria, its activity is not well defined. Here, we developed a bioreporter in *Pseudomonas* sp. FF2 (PFF2) to monitor *alkB* promoter activity in vitro and on *Arabidopsis thaliana* leaves. Single‐cell analysis revealed a highly heterogeneous *alkB* promoter activity, with a subpopulation exhibiting strong fluorescence, consistent with alkane metabolism bet‐hedging. On leaves, the promoter was active over the course of seven days, indicating constant access to alkanes over time. While our results support a potential role of *alkB* in bacterial adaptation to the phyllosphere, direct evidence of cuticular wax degradation is missing. Thus, future studies should trace the incorporation of plant‐derived aliphatic compounds to elucidate the ecological relevance of *alkB* during leaf colonisation.

## Graphical abstract

Given that leaf cuticular waxes restrict access to nutrients and water to bacteria on leaf surfaces, we developed a bioreporter in *Pseudomonas* sp. FF2 to monitor the activity of the *alkB* promoter, involved in alkane degradation. The bioreporter revealed heterogeneous *alkB* expression in vitro and on *Arabidopsis thaliana* leaves, consistent with a bet‐hedging strategy.

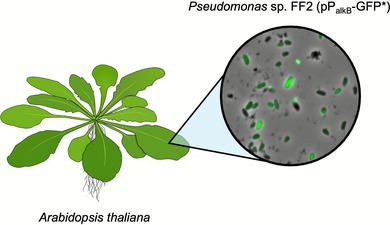

## 1 Introduction

The phyllosphere represents the microbial habitat that is formed by the surfaces of aboveground plant organs, such as stems, flowers, fruits and leaves (Sohrabi et al. 2023). Leaves are the most prevalent part of the phyllosphere and usually, the term phyllosphere is used synonymously with the leaf surface habitat (Leveau 2019). On the macroscale, factors such as UV radiation, fluctuating temperatures, and variable weather conditions make the leaf surface an extreme environment to colonise. On the microscale, challenges include variable nutrient and water availability (Schlechter et al. 2019), largely influenced by the diverse and highly hydrophobic structure of the plant cuticle on the surfaces (Schreiber 2010; Yeats and Rose 2013). The cuticle is primarily composed of cutin, a biopolyester composed of hydroxy‐ and hydroxyl epoxy‐fatty acids, and waxes that include aliphatic compounds, with a highly diverse mixture of carbon chains ranging from C16 to C32 (Schreiber and Schönherr 2009).

Despite these challenges, the phyllosphere is home to many microorganisms, predominantly bacteria, which colonise this environment at densities of 10^6^ and 10^7^ cells per cm^2^ of leaf surface (Schlechter et al. 2019). Bacteria in the phyllosphere have evolved various adaptation strategies to withstand these adverse conditions. Examples include UV protection via pigmentation and DNA repair mechanisms (Schlechter et al. 2019), motility and biosurfactant production (Kunzler et al. 2024), and resource uptake (Delmotte et al. 2009; Müller et al. 2016; Schäfer et al. 2023). As nutrients are a limiting growth factor on the leaf surface, specialised mechanisms for nutrient acquisition represent a fitness advantage, such as sugars and methanol utilisation (Mercier and Lindow 2000; Delmotte et al. 2009; Yurimoto et al. 2021). Many bacteria colonising the phyllosphere have been found to be able to degrade hydrocarbons (Oso et al. 2019). This trait is in most cases linked to the *alkBFGHIJKL* and *alkST* operons (Grund et al. 1975; Nieder and Shapiro 1975), controlled by the AlkS transcription factor. AlkS activates the *alkB* promoter (P*alkB*) in the presence of alkanes. The activity of P*alkB* depends on the availability of alkanes in the growth medium and is therefore well suited as a proxy for the activity of *alkB* (Yuste et al. 2000). The *alkB* gene is one of the most ubiquitous genes involved in alkane degradation, is widely distributed in many phylogenetically diverse bacteria, and is a marker gene of the alkane degradation pathway (Smith et al. 2013). The *alkB* gene codes for a membrane‐bound non‐heme diiron alkane 1‐monooxygenase, involved in the first step of an oxidation pathway to transform alkanes into fatty acids (Guo et al. 2023). Given the ubiquity of *alkB* among phyllosphere‐associated bacteria, it is hypothesised that AlkB‐mediated alkane degradation offers a fitness advantage for bacteria in this environment (Gandolfi et al. 2017).

Certain phyllosphere‐associated bacteria can grow on diesel, including several *Pseudomonas* spp., which are chemically similar to the hydrocarbons found in the cuticle (Oso et al. 2021, 2019). One of these Pseudomonads, *Pseudomonas* sp. FF2 (PFF2), isolated from romaine lettuce (Burch et al. 2016), has been shown to carry the *alkB* gene, utilise diesel as its sole carbon source, and grow on *Arabidopsis thaliana* (Oso et al. 2021). PFF2 is part of the *P. fluorescens lineage* and subgroup and is closely related to *P. extremaustralis* . In this study, we hypothesised that PFF2 expresses *alkB* on plant leaves to degrade cuticular alkanes. To that end, a whole‐cell bioreporter was developed by constructing a plasmid containing a green fluorescent protein (GFP) gene under the native *alkB* promoter of PFF2 to monitor the *alkB* gene activity during leaf surface colonisation. The functionality of the bioreporter was tested in vitro and on *Arabidopsis thaliana* leaves, and single‐cell fluorescence was analysed to provide insights into the activity of the *alkB* promoter in the phyllosphere.

## 2 Experimental Procedures

### 2.1 Strains, Culture Media and Growth Condition

Strains were routinely grown in lysogeny broth (LB (Luria/Miller), Carl Roth) or lysogeny broth agar (LBA (Luria/Miller), Carl Roth). Unless stated otherwise, *Escherichia coli* (ST18) was grown at 37°C and was used for cloning and as a donor for conjugation experiments, while the focal strain *Pseudomonas* sp. FF2 (PFF2) was grown at 30°C. Wherever appropriate, 50 μg mL^−1^ kanamycin (Km, Carl Roth) was used in selective media.

Bushnell Haas Broth (BHB, 409.6 mg/L MgSO_4_·7H_2_O, 26.5 mg/L CaCl_2_·2H_2_O, 1.0 g/L KH_2_PO_4_, 1.0 g/L K_2_HPO_4_, 1.0 g/L NH_4_NO_3_, 83.3 mg/L FeCl_3_·6H_2_O, pH 7.0) was used as a base medium for in vitro growth in diesel. BHB was supplemented with 1% v/v diesel (commercial diesel, locally sourced). A chemical analysis of this diesel was not performed; therefore, its composition was not determined.

### 2.2 Alkane Bioreporter Construction and Conjugation Into PFF2

To monitor *alkB* promoter activity, we constructed the plasmid pFru97‐PalkB‐mClover3‐[AAV] (pPalkB‐GFP*), which expresses a destabilised version of the green fluorescent protein mClover3 under the control of a 300‐bp native *alkB* promoter fragment from *Pseudomonas* sp. FF2 (PFF2) (Figure S1). The reporter cassette was assembled via isothermal assembly and inserted into the broad‐host‐range vector pFru97 (Supporting Information). The assembled pPalkB‐GFP* plasmid was then transformed into NEB Turbo competent *E. coli* cells (New England Biolabs) according to the manufacturer's recommendations and was selected on LBA + Km.

The pPalkB‐GFP* plasmid was used to transform *E. coli* ST18, an auxotrophic donor strain for conjugation, on LBA supplemented with Km + 5‐aminolevulinic acid (50 mg/mL) (Thoma and Schobert 2009). Following ST18 transformation, the pPalkB‐GFP* plasmid was transferred into PFF2 via biparental mating (Supplemental Methods) (Schlechter et al. 2019). The resulting strain, PFF2_P*alkB*‐GFP*_, was used for all subsequent experiments.

### 2.3 Diesel Utilisation Assay and P*alkB* Activity in Bushnell‐Haas Broth and LB

Overnight cultures of PFF2_P*alkB*‐GFP*_ grown in LB + Km at 30°C were used to inoculate BHB medium supplemented with 1% v/v diesel (Oso et al. 2021). The overnight cultures were washed by centrifugation for 10 min at 5000 × *g* and then resuspended in 5 mL BHB (*N* = 3). PFF2_P*alkB*‐GFP*_ was grown in 50 mL BHB with or without 1% v/v diesel. BHB + 1% v/v diesel without bacteria was used as a negative control. All cultures were supplemented with Km and incubated at 30°C with constant shaking at 300 r.p.m. Optical density at 600 nm was measured using a spectrophotometer (GeneQuant 100, Biochrom) in three to four‐day intervals over a total time of 28 days. At days 1, 6, and 28, aliquots were sampled and prepared for microscopy. Furthermore, the fluorescence intensity of PFF2_P*alkB*‐GFP*_ was measured in LB.

### 2.4 Growth of PFF2_P*alkB*_ _‐GFP*_ *in Planta* and Activity of P*alkB*

To determine *alkB* promoter activity during colonisation of *A. thaliana* leaves, PFF2_P*alkB*‐GFP*_ was inoculated onto axenically grown *A. thaliana* Col‐0 (Supplemental Methods), as described in (Miebach et al. 2020). Plants were grown in Magenta GA‐7 (Merck) containing zeolite (Zeolith‐100, Steinlando) supplemented with ¾ Murashige and Skoog (MS with vitamins, pH 5.8, Duchefa). Boxes were placed in a plant growth cabinet (poly klima GmbH) at 21°C in an 11/13 h photoperiod, with a light intensity of ~120 μE m^−2^ s^−1^, and 80% relative humidity. Four‐week‐old plants were inoculated with an exponentially grown PFF2_PalkB‐GFP*_ culture (OD_600nm_ = 0.05 in PBS) using an airbrush sprayer (Ultra airbrush, Harder & Steenbeck GmbH & Co. KG; compressor, Sparmax TC‐620X airbrush compressor). The inoculated boxes were then placed back into the plant growth cabinet under the same controlled conditions.

Whole *A. thaliana* rosettes were sampled on day zero, two, and seven after inoculation, with six to eight biological replicates per sampling point. PBS was added to the samples, vortexed for 10 s, sonicated for 5 min at 75% intensity in a sonication bath (Emmi‐12HC, EMAG AG), and then vortexed for an additional 15 s. An aliquot of the leaf wash was used to prepare a tenfold dilution series to determine colony‐forming units (CFU) on LBA + Km. Plant fresh weight was recorded for normalization of colony counts. The remaining suspension was used for microscopy.

### 2.5 Microscopy

Bacterial suspensions were centrifuged at 15,000 × *g* for 10 min at 4°C, with the pellet resuspended in 50 μL of 4% paraformaldehyde solution (PFA, 4% w/v PFA in 1 × PBS w/v, pH 7.2). Samples were incubated for 1 h at room temperature or overnight at 4°C. Cells were washed three times by centrifugation at 15,000 × *g* for 5 min at 4°C and resuspended each time in PBS. After the final wash, the pellet was resuspended in 50 μL PBS. Subsequently, 50 μL ethanol was added to the samples for storing at −20°C. Samples were processed within 24 h after harvesting.

Microscopy was performed on an Axio Imager.Z2 (Carl Zeiss) using an EC Plan‐Neofluar 100 × /1.3 oil Ph3 objective using phase contrast and the Zeiss Filter 38 HE (BP 470/40, FT 495, BP 525/50), to acquire phase contrast images and mClover3 signals, respectively. Micrographs were acquired using an Axiocam 712 mono camera (Carl Zeiss) and the programme ZEN Blue 3.3 (Carl Zeiss).

### 2.6 Image Processing

Quantitative image analysis was performed using custom macros to automate image processing in FIJI v. 2.3.0 (Schindelin et al. 2012). Raw images were imported and converted to 8‐bit grayscale. A training data set was generated from random images for cell classification. To that end, phase contrast images were used for segmentation with a threshold applied based on the image type (in vitro or *in planta* images), set between one and four standard deviations below the mean pixel intensity. Masks were created and refined using morphological operations such as watershed segmentation, erosion, and dilation. Particles between 0.25 and 6.0 μm^2^ were detected, and each particle was manually classified as a bacterial cell or non‐cell. The data were then used in machine learning model training.

For automated cell segmentation, images were converted to 8‐bit grayscale, and phase contrast and green fluorescent channels were extracted. A mask was generated from the phase contrast as explained above. Particles were selected and mapped onto the green fluorescent channel for fluorescence intensity measurement. Additional measurements were recorded for cell classification using machine learning, including area, mean pixel intensity, perimeter, shape descriptors (major and minor axis, angle), circularity, Feret's diameter (Feret, FeretX, FeretY, Feret Angle, MinFeret), aspect ratio, roundness, and solidity.

Background fluorescence was estimated from images that were thresholded using the percentile method. The resulting binary mask was inverted to exclude regions with bacterial biomass. Then, 50 randomly positioned 2 × 2‐pixel squares were selected across the background regions, and the mean fluorescence intensity in the green, fluorescent channels was measured. These values were used for downstream fluorescence correction.

### 2.7 Machine Learning Model Development

Cell classification was performed using machine learning, with models trained on ground truth‐labelled datasets. Using a custom macro script in FIJI/ImageJ, particles from a subset of images were manually classified as cells or non‐cells, and particle descriptors were recorded to identify the most relevant predictors for classifying real datasets. Two machine learning models for each of the in vitro and *in planta* datasets were trained to classify single cells: a logistic regression (LR) with L1 regularisation, and a random forest (RF) model. These models were developed using the R package *tidymodels* (Kuhn and Wickham 2020). The LR model was implemented using the *glmnet* engine with an L1 penalty (Lasso regression), with the penalty parameter optimised over a logarithmic spaced grid from 10^−4^ to 10^−1^. The best penalty was then used to improve the model based on the higher area under the receiver operating characteristic (ROC) curve. The RF model was implemented using the *ranger* engine with 500 trees. The number of predictors sampled at each split and the minimum number of observations required in a node were tuned over 25 combinations, selecting the best parameters based on ROC performance. Single‐cell data was split into two datasets: training (75%) and test (25%). Both models were trained using ten‐fold cross‐validation on the training data set, and the final models were evaluated on the test dataset. Performance metrics were estimated, including accuracy, precision, recall, F1‐score, kappa statistics, area under the ROC curve (ROC‐AUC), sensitivity, and specificity. Feature importance was assessed using variable importance plots to select the best models.

### 2.8 Single‐Cell Fluorescence Analysis

Fluorescence of single cells was analysed in R (R Core Team 2021). Cells classified as positive by the best‐performing machine learning model were retained for further analysis. Fluorescence values were corrected by subtracting the median background intensity from each cell's fluorescence signal per image, and replicates with insufficient cell counts were excluded.

The distribution of fluorescence values was assessed using normal probability plots, comparing the observed fluorescence data with an expected normal distribution. Deviations from normality were evaluated using skewness and kurtosis, calculated with the *moments* package (Komsta and Novomestky 2022). A normal distribution was defined by skewness = 0 and kurtosis = 3. Positive skewness indicates a right‐tailed distribution, while kurtosis > 3 suggests the presence of heavy tails (extreme values).

To quantify high‐fluorescent cells and determine fold change relative to a control, a baseline fluorescence threshold was calculated using the interquartile range criterion, defined as I = *Q*_75_ + 1.5 × IQR, where *Q*_75_ was the 75th percentile of fluorescence values from BHB‐grown cells without diesel (in vitro), or time zero (*in planta*).

Statistical analyses were performed using the *rstatix* package (Kassambara 2023) at = 0.05, including normality tests (Shapiro–Wilk), homoscedasticity tests (Levene's), and comparisons between fluorescence distributions using t‐tests, ANOVA, and Tukey's HSD post hoc tests for multiple comparisons. Kruskal‐Wallis and Dunn's tests were used as non‐parametric alternatives. Results were visualised in cumulative distribution and probability plots, as well as comparisons of the highest fluorescence values across time points.

## 3 Results

### 3.1 PFF2 Growth *alkB* Promoter Activity in Vitro

**FIGURE 1:**
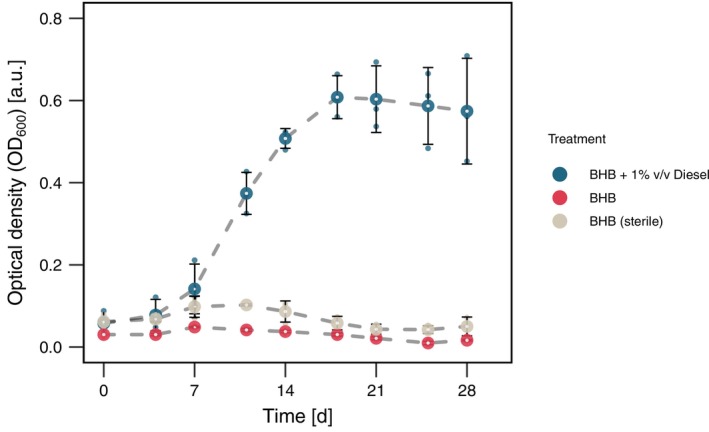
Growth of PFF2_P*alkB*‐GFP*_ in diesel‐supplemented media. Optical density (OD_600_) measurements of *PFF2PalkB‐GFP* grown in Bushnell‐Haas broth (BHB) supplemented with 1% v/v diesel, compared to BHB without a carbon source and a non‐inoculated sterile control. Growth was monitored over time to assess the impact of diesel as a sole carbon source.

To investigate the growth of PFF2_P*alkB*‐GFP*_ and the *alkB* promoter activity in diesel‐supplemented minimal media, we cultured PFF2_P*alkB*‐GFP*_ in BHB with 1% v/v diesel and in BHB without an added carbon source. PFF2_P*alkB*‐GFP*_ reached a mean maximal OD_600nm_ of 0.61 ± 0.052 after 18 days (Figure 1). By contrast, growth was negligible in BHB alone, with no change in OD_600nm_. The increase in OD_600_ correlated with elevated *alkB* promoter activity, as measured by single‐cell fluorescence imaging. A trained random forest model classified individual cells with high accuracy, precision, and ROC AUC (> 90%), outperforming a logistic regression model marginally (Figure S2A). The most important classification features were the growth condition (BHB no diesel), particle solidity (area/convex area ratio), and aspect ratio (Figure S2B,C).

Fluorescence distributions of PFF2_P*alkB*‐GFP*_ were consistently right‐skewed across all conditions (Figure S3). In diesel‐grown PFF2_P*alkB*‐GFP*_, skewness and kurtosis averaged 2.7 and 25.2, respectively, compared to 2.1 and 8.1 in non‐diesel supplemented media. This strong positive skewness (> 2) and kurtosis (> 3) reflected a large proportion of low‐fluorescence cells. Over time, 46%–90% of diesel‐grown PFF2_P*alkB*‐GFP*_ cells showed fluorescence values below 10% of the maximum measurable intensity, compared to 66%–83% in cells grown in BHB without diesel. As a comparison, 90% of cells grown in LB displayed low fluorescence values; however, maximal values were comparable to cells grown in BHB without diesel. This consistent pattern across conditions suggests that heterogeneity in *alkB* promoter activity is intrinsic to a population, regardless of growth condition.

**FIGURE 2:**
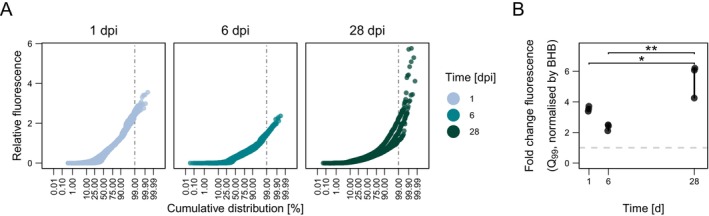
Relative fluorescence distribution and high‐intensity cell subpopulations of PFF2_P*alkB*‐GFP*_ in diesel‐supplemented media over time. (A) Normal probability plot of PFF2_P*alkB*‐GFP*_ single‐cell green fluorescence in BHB + 1% diesel at 1, 6, and 28 days, relative to baseline fluorescence. Baseline fluorescence was defined using the interquartile range (IQR) criterion (I = Q3 + 1.5 × IQR) for cell populations incubated in BHB without diesel at each time point. The vertical line marks the 99th percentile of the fluorescence distribution. (B) Fold change in fluorescence of the top 1% of cells (99th percentile), normalised to baseline fluorescence. The horizontal line indicates a fold change of 1. Asterisks (*) denote statistically significant differences among groups (*p* < 0.05, ANOVA with Tukey's post hoc test).

To further assess fluorescence dynamics, we normalized fluorescence in diesel‐grown cells to the fluorescence of cells grown in BHB alone medium at each time point. Median relative fluorescence remained low across time points (0.23–0.46‐fold), but skewness and kurtosis increased especially at day 28 (Figure 2A, Table 1). However, at one day post inoculation, 19% of cells exceeded baseline fluorescence, with some cells reaching up to 3.6‐fold higher fluorescence. By day six, only 3.9% of cells exhibited higher fluorescence, with a maximum of 2.4‐fold above baseline. By day 28, relative fluorescence increased, with 11% of cells exceeding baseline fluorescence by up to 5.8‐fold.

**TABLE 1:** Summary of in vitro single cell microscopy of PFF2 grown in BHB + 1% diesel.

Time [dpi]	Biological replicates	*N* cells	Median rel. fluorescence	IQR rel. fluorescence	Proportion high fluorescence [%]	Max rel. fluorescence	Skewness	Kurtosis
1	3	3839	0.46	0.122	18.5	3.6	1.44	5.93
6	3	5721	0.23	0.055	4.6	2.4	1.46	5.83
28	3	10,988	0.23	0.067	3.78	5.8	3.30	24.5

To determine whether highly fluorescent cells differed significantly from the overall populations, we analysed the top 1% of fluorescent cells across growth conditions (Figure 2B). The median relative fluorescence of these cell subpopulations was higher than baseline at every sampling point (one‐sample t‐test, *p* < 0.05). Differences between time points were observed (ANOVA, *F*(2,6) = 18.43, *p* = 0.003), with cell fluorescence at day one and six being statistically lower than at day 28 (Tukey's HSD, *p* < 0.05).

Taken together, these results indicate that while diesel supplementation supports the growth of PFF2, *alkB* promoter activity is highly heterogeneous within the population. A small fraction of cells exhibited elevated fluorescence, particularly at early and late time points, while fluorescence was low in most cells. Thus, only a subset of cells actively engaged in alkane degradation at a given time.

### 3.2 Growth and Promoter Activity on Arabidopsis

**FIGURE 3:**
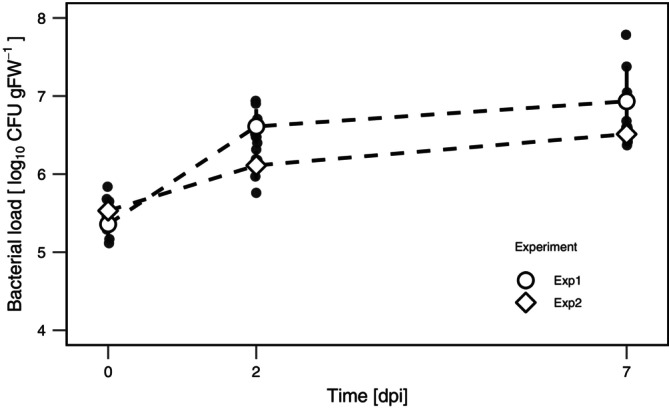
Population growth of PFF2_P__*alkB*‐GFP*_ on *A. thaliana* . Bacterial loads were quantified as CFU per gram of fresh weight across two independent experiments. (*N* = 8 biological replicates per experiment).

We assessed the dynamics of population growth and single‐cell fluorescence over time on *A. thaliana* to evaluate the heterogeneity of PFF2_P*alkB*‐GFP*_ in the phyllosphere. Two independent experiments showed a consistent pattern of population growth (Figure 3). At time zero, median CFU values ranged from 2.07 to 3.30 × 10^5^ CFU per gram of leaf (gFW). By day two, CFU increased 6‐ to 20‐fold, reaching 1.40–4.15 × 10^6^ CFU gFW^−1^, and maintaining high levels by day seven, with a median of 3.38–6.35 × 10^6^ CFU gFW^−1^.

In parallel, we sampled bacterial cells and assessed their fluorescence as a proxy for *alkB* promoter activity using fluorescence microscopy. Similar to the in vitro dataset, a random forest model slightly outperformed a logistic regression model and was therefore used for cell classification (Figure S4A). The most important classification features included particle solidity, aspect ratio, and roundness (Figure S4B,C).

**FIGURE 4:**
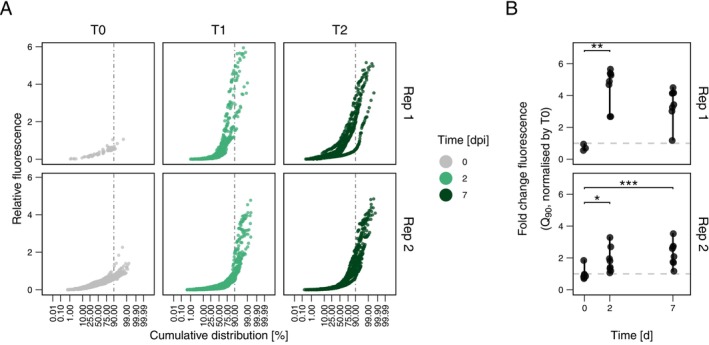
Distribution of relative single‐cell fluorescence and *alkB* promoter activity in PFF2_P*alkB*‐GFP*_ in *A. thaliana*. (A) Normal probability plot of PFF2_P*alkB*‐GFP*_ single‐cell GFP fluorescence at different sampling points (0‐, 2‐, and 7‐days post‐inoculation) relative to the baseline fluorescence. Baseline fluorescence was defined using the IQR criterion (*I* = q.75 + 1.5 × IQR) from cell populations at time zero (0 dpi). The vertical line indicates the fluorescence threshold below which 90% of the cell population falls. (B) Fold change in fluorescence of the top 10% most fluorescent cells (90% quantile), normalised to the baseline fluorescence. The horizontal line represents a fold change of 1. Asterisks indicate statistically significant differences among groups (* = *p* < 0.05, ** = *p* < 0.01, *** = *p* < 0.001; Kruskal‐Wallis test with Dunn's post hoc and Holm‐Bonferroni correction).

Single‐cell data showed a consistent pattern of increasing fluorescence heterogeneity over time across the two independent experiments. Fluorescence distributions became more right‐skewed and had high kurtosis (Figure S5). To evaluate changes in *alkB* promoter activity, we normalized the fluorescence values to the fluorescence of cells at time zero (Figure 4A). At the time of inoculation, cell fluorescence showed the lowest skewness and kurtosis (Table 2), indicating a near‐normal distribution. By day two, the distribution of single‐cell fluorescence became more asymmetrical, with increased skewness and kurtosis (Table 2). This pattern persisted on day seven, revealing consistent heterogeneous P_*alkB*_ activity. Within the cell populations, the proportion of highly fluorescent cells increased over time, with 8.2%–36.1% of cells exceeding baseline fluorescence by day two and 16.9%–36.2% by day seven.

**TABLE 2:** Summary of *in planta* single‐cell microscopy.

Exp.	Time [dpi]	Biological replicates	*N* cells	Median rel. fluorescence	IQR rel. fluorescence	Proportion high fluorescence [%]	Skewness	Kurtosis
1	0	5	55	0.35	0.12	0.0	0.45	1.78
	2	8	428	1.34	0.60	36.1	1.30	3.51
	7	8	1172	1.07	0.28	36.2	1.75	5.92
2	0	8	761	0.39	0.11	2.1	1.69	6.63
	2	8	1392	0.37	0.05	8.2	3.44	16.3
	7	8	2113	0.55	0.18	16.9	2.69	10.6

To further dissect these changes in *alkB* promoter activity, we analyzed the top 10% most fluorescent cells and compared their fluorescence relative to baseline levels (Figure 4B). In both experiments, median relative fluorescence of this subpopulation was higher than baseline at day two (one‐sample t‐test, experiment 1: *t*(7) = 8.36, *p* = 6.9 × 10^−5^; experiment 2: *t*(7) = 3.03, *p* = 0.0191) and day seven (experiment 1: *t*(7) = 6.56, *p* = 3.2 × 10^−4^; experiment 2: *t*(7) = 4.84, *p* = 1.9 × 10^−3^). Relative fluorescence also varied between time points (Kruskal‐Wallis, *H*(2) = 2, *p* = 0.368), with significantly higher fluorescence at day two compared to time zero (Dunn's test, *p*_*Exp1*_ = 0.00612; *p*_*Exp2*_ = 0.0197). Additionally, in the second experiment, the median relative fluorescence increased twofold on day seven compared to time zero (Dunn's test, *p =* 0.00235). This analysis confirmed that while median fluorescence of the total population remained low, a subset of highly fluorescent cells maintained or even increased their fluorescence relative to baseline levels.

Overall, these results showed that PFF2_P*alkB*‐GFP*_ single‐cell fluorescence heterogeneity increased in the phyllosphere over time. While median fluorescence was low, subpopulations of highly fluorescent cells persisted, suggesting that *alkB* promoter activation is differentially regulated within a population.

## 4 Discussion

The phyllosphere is a nutrient‐limited environment where access to easily available carbon sources such as sugars is heterogeneous (Leveau and Lindow 2001; Remus‐Emsermann et al. 2011). Long‐chain alkanes in plant cuticles may represent an alternative carbon pool, and their composition varies between plant species (Fernández et al. 2016), and these differences affect the structure of leaf‐associated bacterial communities (Bodenhausen et al. 2014; Ritpitakphong et al. 2016). The leaf‐associated *Pseudomonas* sp. PFF2 strain used in this study carries the alkane 1‐monooxygenase gene *alkB* and its presence has been shown to be essential for growth when alkanes such as those present in diesel are the sole carbon source (Oso et al. 2021).

Using our single‐cell bioreporter, we show that *alkB* promoter activity is inducible in vitro when diesel is the sole carbon source but its expression remains highly heterogeneous, with most cells showing low fluorescence and a small fraction strongly activating the *alkB* promoter. Over time, 46%–90% of diesel‐grown cells exhibited fluorescence below 10% of the maximum measurable intensity, supporting non‐uniform *alkB* activation. Such heterogeneity suggests a potential bet‐hedging strategy, where only a subset of cells invest in alkane metabolism while others persist in alternative metabolic states or scavenge metabolites released by *alkB*‐expressing cells (Kamrad et al. 2023). In LB and BHB without diesel, few cells still showed higher‐than‐expected fluorescence, suggesting that *alkB* expression is not fully repressed under non‐alkane conditions, possibly due to stochastic gene expression or other regulatory mechanisms.

PFF2, though originally isolated from lettuce, can sustain growth on *A. thaliana* leaves, as shown here and in previous work (Oso et al. 2021). Our reporter showed that most cells maintained low *alkB* promoter activity, while a subpopulation showed increased activity over seven days. This pattern may reflect initial use of easily available sugars followed by a shift toward alkanes as sugars are depleted. The resulting fluorescence heterogeneity is consistent with the patchy chemical landscape of leaf surfaces (Remus‐Emsermann and Schlechter 2018), where local variation in the availability of the cuticle and other nutrients likely contributes to the heterogeneous *alkB* expression pattern.

Our observations do not demonstrate direct cuticle degradation or alkane assimilation but show that *alkB* can be expressed during leaf colonisation, potentially providing an alternative option when preferred carbon sources become scarce. Further work combining fluorescence in situ microscopy and multi‐bioreporter strains could clarify how highly fluorescent cells are spatially distributed on leaves and how alkane metabolism integrates with other survival strategies in the leaf environment.

## 5 Conclusion

Previous studies have shown that bacteria carrying *alkB* genes are prevalent on leaf surfaces. However, the role of *alkB* in utilizing alkanes and other aliphatic components from leaf cuticles remains unclear. Here, we have provided further evidence that subpopulations of bacteria on leaves can activate *alkB* expression, indicating its role in resource utilization when other nutrients may be scarce. However, direct functional evidence of cuticular wax degradation by phyllosphere bacteria is still missing. Future experiments should focus on this in further detail, for example, by measuring the incorporation of radiolabelled cuticular waxes during bacterial colonization of leaves and the activity of other proxy genes for aliphatic compounds, such as *almA*. Given the complexity in the interactions of microbes with leaf surfaces, further work is needed to establish how alkane metabolism and cuticular wax utilization influence bacterial adaptation across plant species.

## Author Contributions

**Mitja Remus‐Emsermann:** conceptualization, investigation, funding acquisition, writing – review and editing, writing – original draft, methodology, project administration, supervision, resources. **Rudolf Schlechter:** conceptualization, investigation, writing – review and editing, visualization, methodology, validation, formal analysis, supervision, data curation. **Laura Voß:** investigation, writing – original draft, visualization, methodology, formal analysis, resources. **Evan J. Kear:** writing – review and editing, methodology, resources, validation, investigation. **Mila Oeltjen:** resources, validation, investigation.

## Conflicts of Interest

The authors declare no conflicts of interest.

## Supporting information


**Figure S1:** Plasmid construction. The *alkB* promoter of *Pseudomonas* sp. FF2 and green fluorescence protein gene mClover3‐[AAV] were amplified and inserted into the backbone of the HindIII‐digested pFru97 expression plasmid through Gibson assembly, resulting in plasmid pFru97‐alkB‐mClover‐[AAV] (pPalkB‐GFP*).
**Figure S2:** Comparison of supervised machine learning models for in vitro single‐cell data analysis. (A) Performance metrics of logistic regression (LR) and random forest (RF) models for cell classification of PFF2 cells grown in vitro (BHB or LB medium). Feature importance of (B) LR and (C) RF models. Features: Treatment_X1 (BHB medium); Treatment_X2 (LB medium); Minor (minor axis length); Major (major axis length); MinFeret (minimum feret diameter); Feret (maximum feret diameter); AR (aspect ratio); Round (roundness); Circ. (circularity); Solidity (area/convex hull area ratio); Area (total number of pixels within a particle).
**Figure S3:** Distribution of PFF2_PalkB‐GFP*_ single‐cell GFP fluorescence in vitro. Normal probability plot of cell fluorescence of PFF2_PalkB‐GFP*_ grown in BHB supplemented with or without diesel over time (1, 6, and 28 days) or LB. Vertical line indicates the 99% of cells. Single‐cell fluorescence of PFF2_PalkB‐GFP*_ grown in LB fluorescence was included for comparison (grey diamond).
**Figure S4:** Comparison of supervised machine learning models for *in planta* single‐cell data analysis. (A) Performance metrics of logistic regression (LR) and random forest (RF) models for cell classification of PFF2 cells grown in planta ( *A. thaliana* phyllosphere). Feature importance of (B) LR and (C) RF models. Features: Minor (minor axis length); Major (major axis length); Perim. (perimeter); MinFeret (minimum feret diameter); Feret (maximum feret diameter); FeretAngle (angle of the maximum feret diameter); AR (aspect ratio); Round (roundness); Circ. (circularity); Solidity (area/convex hull area ratio); Area (total number of pixels within a particle).
**Figure S5:** Distribution of PFF2_PalkB‐GFP*_ single‐cell GFP fluorescence in the *Arabidopsis phyllosphere*. Normal probability plot of PFF2_PalkB‐GFP*_ single‐cell GFP fluorescence at different sampling points (0‐, 2‐, and 7‐days post‐inoculation). Each point represents the fluorescence intensity of an individual cell. Fluorescence at time zero is included in every plot as a reference (grey).

## Data Availability

The data that support the findings of this study are openly available in Zenodo at https://doi.org/10.5281/zenodo.15014570 (Voß et al. 2025). The R scripts used for data analysis are accessible in the following GitHub repository: https://github.com/relab‐fuberlin/alkanebioreporter.
